# A Miniature Multi-Functional Photoacoustic Probe

**DOI:** 10.3390/mi14061269

**Published:** 2023-06-19

**Authors:** Riqiang Lin, Jiaming Zhang, Wen Gao, Xiatian Wang, Shengmiao Lv, Kwok-Ho Lam, Xiaojing Gong

**Affiliations:** 1 Department of Electrical Engineering, The Hong Kong Polytechnic University, Hung Hom, Kowloon, Hong Kong, China; riqiang33.lin@connect.polyu.hk (R.L.);; 2 Research Center for Biomedical Optics and Molecular Imaging, Shenzhen Key Laboratory for Molecular Imaging, Guangdong Provincial Key Laboratory of Biomedical Optical Imaging Technology, Shenzhen Institute of Advanced Technology, Chinese Academy of Sciences, Shenzhen 518055, China; 3 Centre for Medical and Industrial Ultrasonics, James Watt School of Engineering, University of Glasgow, Glasgow G12 8QQ, UK

**Keywords:** miniature probe, transparent ultrasound transducer, GRIN lens backing, OR-PAM

## Abstract

Photoacoustic technology is a promising tool to provide morphological and functional information in biomedical research. To enhance the imaging efficiency, the reported photoacoustic probes have been designed coaxially involving complicated optical/acoustic prisms to bypass the opaque piezoelectric layer of ultrasound transducers, but this has led to bulky probes and has hindered the applications in limited space. Though the emergence of transparent piezoelectric materials helps to save effort on the coaxial design, the reported transparent ultrasound transducers were still bulky. In this work, a miniature photoacoustic probe with an outer diameter of 4 mm was developed, in which an acoustic stack was made with a combination of transparent piezoelectric material and a gradient-index lens as a backing layer. The transparent ultrasound transducer exhibited a high center frequency of ~47 MHz and a −6 dB bandwidth of 29.4%, which could be easily assembled with a pigtailed ferrule of a single-mode fiber. The multi-functional capability of the probe was successfully validated through experiments of fluid flow sensing and photoacoustic imaging.

## 1. Introduction

Photoacoustic technology, as a hybrid biomedical imaging modality [1,2], is capable of providing morphologic and functional information from cells to organs [3,4]. Its principle is that a short-pulsed laser beam irradiates the region of the biological tissue. The tissue absorbs the laser energy, inducing an instantaneous temperature rise and transient thermoelastic expansion. Additionally, ultrasound—also called photoacoustic waves—is then generated from the tissue. To ensure efficient irradiation and detection, the strategy of developing a coaxial construction of optical–acoustic beams and beam focusing is the warranted measure for probe design [5], such as optical-resolution photoacoustic microscopy (OR-PAM) [6,7,8]. As an essential element of system, lead zirconate titanate (PZT) has been used in ultrasound transducers for OR-PAM, which could hinder the laser delivery by its opaque appearance. To improve the system performance, the conventional OR-PAM essentially involves specific optical and acoustic designs [7,9], offering coaxial excitation and detection to overcome the opacity issue of the transducer. However, the transducer design with optical–acoustic prisms [7] is complicated and difficult to fabricate, making the imaging probe bulky and heavy.

To combine the ultrasound and optical beams coaxially, some transparent ultrasound transducers (TUTs) have been developed in recent years [10,11,12,13,14], providing solutions with flexibility on the PAM including the quadruple system [15]. Though lithium niobate (LN), one type of transparent piezoelectric single crystal, was commonly selected to develop the TUTs in the literature [10,11,14], there are still some challenges in the development of photoacoustic probes. First, the dimensions of most reported probes were ~1 cm. The LN wafer should be connected with electrical wires using conductive glue that adversely affects the transparency of the TUT. As for high-frequency transducers, the smaller aperture of the wafer would allow less room for electrical connection, making the fabrication much more challenging. Secondly, indium tin oxide (ITO) is usually coated on the LN wafer as transparent electrodes of TUTs. In general, the thinner ITO coating offers better transparency but higher electrical resistance, causing an inefficient operation of the TUT. Thirdly, optical lenses used for focusing lasers in the reported work were separated with the TUTs such that a mount must be applied to fix and adjust the lens coaxially with the TUT. Therefore, the TUT with the lens mount would give a bulky photoacoustic probe, restricting the applications especially for those requiring a compact portable setup [16,17], such as in vivo photoacoustic flow cytometry [18,19,20,21] and wearable PAM [22,23,24].

To simplify the manufacturing process and miniaturize the probe, we present here the design and construction of a 4 mm outer-diameter photoacoustic imaging probe prototype, which has the smallest dimension in the reported work on the TUTs fabricated using the LN single crystal. A miniature 47 MHz TUT was fabricated using the LN wafer with the dimensions of 2 mm × 2 mm × 75 μm. For the first time, a gradient-index (GRIN) lens was employed for beam shaping and as the backing layer of the TUT, simplifying the probe structure. In addition, the weight of the entire TUT was only ~0.3 g, which was light enough to be positioned on the skin without causing an uncomfortable feeling. Compared with the previous works, the novel design significantly shrinks the dimension of the transparent photoacoustic probe and greatly improves the working frequency of LN-based TUTs. Moreover, the design allows the ease of installation and replacement of single-mode (SM) fibers for imaging using different laser wavelengths. The multi-functionality of the probe was validated by experiments of fluid flow sensing and photoacoustic imaging, showing promising potential for various biomedical application scenarios.

## 2. Materials and Methods

### 2.1. Fabrication of TUT

**Figure 1:**
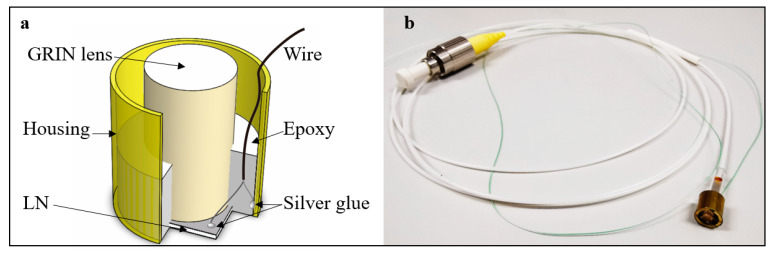
Schematic and photograph of a photoacoustic probe. (**a**) Structure of the probe; (**b**) Photograph of the miniature probe prototype.

A 10 mm × 10 mm LN wafer (Shanghai Bontek optoelectronic technology development Co. Ltd, Shanghai, China) was selected and polished to 75 μm, and then coated with 150 nm thick ITO on both surfaces. The wafer was cut into small pieces with the dimensions of 2 mm × 2 mm as the piezoelectric layer of the TUT. Figure 1a shows the schematic structure of the TUT. To fabricate the TUT, the LN wafer was first positioned at the bottom of the brass housing using wax. The GRIN lens (GRIN2906, Thorlabs, Newton, NJ, USA) was adhered with the wafer by epoxy (301, Epoxy Technology Inc., Billerica, MA, USA). Secondly, the positive electrode of the wire was connected with the inner wall of the housing, while the negative one was adhered to the back side of the LN wafer using silver glue. Then, epoxy was applied to fix all the movable components. Finally, the silver glue was used to connect with the housing and the front side of the LN wafer after the epoxy was cured. Figure 1b shows the photo of the TUT assembled with the SM fiber.

### 2.2. Setup of System and Photoacoustic Probe

**Figure 2:**
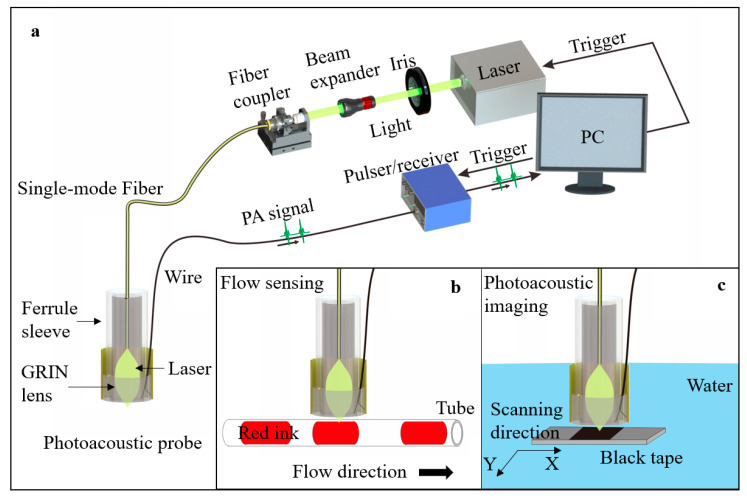
Schematics of system, probe, and experimental setup. (**a**) Three-dimensional drawing of a system with the photoacoustic probe. (**b**) Schematic of flow sensing setup. (**c**) Schematic of photoacoustic imaging setup.

A pulsed Nd:YAG laser (Medical Technology (Shenzhen) Co., Ltd., Shenzhen, China) with 532 nm wavelength was delivered through an iris and a beam expander (EX-532-2X-A, Sanke, Shanghai, China), then coupled into the SM fiber by an objective (PLAN 4X, Shangguang, China). The SM fiber (SMPF0106, Thorlabs, Newton, NJ, USA) with a pigtailed ferrule was applied for laser delivery. The offered laser energy was about 1 μJ. A ferrule sleeve (51-2800-1800, Thorlabs, Newton, NJ, USA) was used to align the GRIN lens and the fiber coaxially. The TUT was used to detect photoacoustic signals from phantoms. The signal was sent to an ultrasound pulser/receiver (5073PR, Olympus, Tokyo, Japan) and then digitized with a DAQ board (ATS9371, Alazar Tech, Pointe-Claire, QC, Canada) in the computer. The schematics of the system, probe, and experimental setup are demonstrated in Figure 2.

### 2.3. Resolution Test

The imaging resolution depends on the laser beam size [7], which is much smaller than the ultrasound detection area. The knife-edge method [25] was used to evaluate the dimension of the laser spot, which has been employed as a standard method for Gaussian laser beam characterization. A blade edge was imaged by the proposed probe. When the laser spot scanned across the blade edge, the change in energy density led to a variation in photoacoustic signals. The beam profile could be characterized by the signals.

### 2.4. Performance Test

To evaluate the acoustic performance of the TUT, a pulse-echo test was conducted. The ultrasound signals were received by the pulser/receiver and then displayed on a digital oscilloscope (DSOX2012A, Keysight, Santa Rosa, CA, USA). An impedance analyzer (4294A, Agilent Tech., Santa Clara, CA, USA) was used for dielectric and electrical impedance measurements at room temperature.

To demonstrate the multi-functionality of the probe, red ink and black tape were selected as targets in the setups of photoacoustic flow cytometry and photoacoustic microscopy, as shown in Figure 2b,c. The red ink in a polyethylene (PE) tube was used for mimicking the blood in the vessel. The red ink was controlled by a pump (HL-2S, Shanghai Huxi, Shanghai, China), flowing at a rate of 20 mm/s.

To evaluate the imaging performance, the photoacoustic probe was fixed on a three-dimensional translation stage to scan the black tape in a water tank. Water was used as the medium of signal transmission. More details can be found in the supplementary materials.

## 3. Results

### 3.1. Performance of TUT

**Figure 3:**
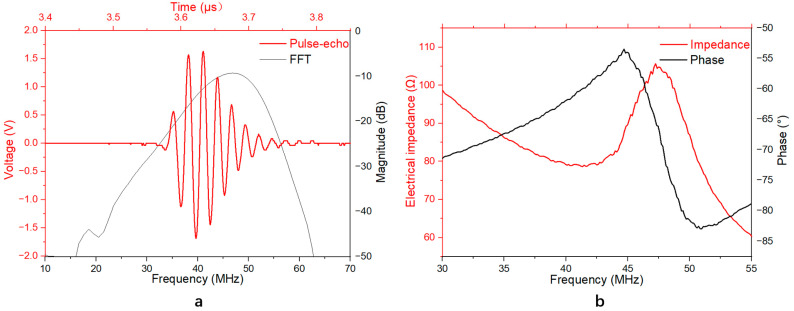
Performance of the TUT. (**a**) Pulse-echo results; (**b**) Impedance/phase frequency spectra.

Figure 3a shows the measured pulse-echo response of the TUT and its Fast Fourier Transform (FFT) spectra without gain. The results show that the measured center frequency of the transducer was 46.9 MHz with a −6 dB bandwidth of 29.4%. Figure 3b shows that the developed TUT exhibited an obvious resonance mode even when the piezoelectric layer was loaded by the GRIN lens, in which the resonant frequency *f_r_* was 41.3 MHz with an electrical impedance of 78 Ω. Compared to the desired electrical impedance (50 Ω), the measured one was slightly higher, which may be due to the imperfect electrical behavior of ITO electrodes.

With an anti-resonant frequency, *f_a_*, of 47.2 MHz, the effective electromechanical coupling coefficient *k_eff_* can be calculated by the following equation:

$$k_{eff}=\sqrt{1-\frac{f_{r}^{2}}{f_{a}^{2}}}$$

The *k_eff_* of the TUT was ~0.48, which is comparable to that of the reported TUT fabricated using LN as the piezoelectric material [11].

### 3.2. Resolution of Photoacoustic Probe

**Figure 4:**
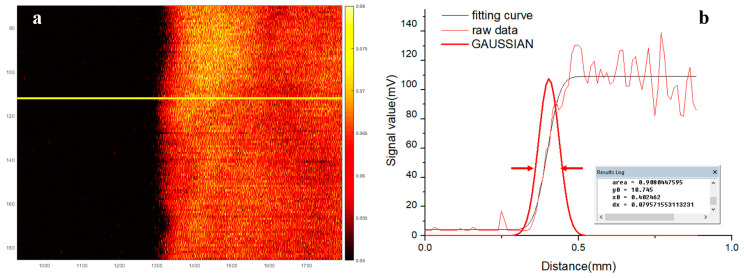
Result of resolution test. (**a**) Photoacoustic image of the blade edge. The yellow line represents the position of the extracted raw data. (**b**) Photoacoustic signal of yellow line in (**a**) and the measured resolution. The red arrows indicate the position of FWHM.

Figure 4 shows the result of the resolution test. Figure 4a shows the photoacoustic image of the blade edge. Figure 4b shows the raw data of the yellow line in Figure 4a and the calculated data, in which the fitting curve is the edge spread function (ESF). The Gaussian curve was the line spread function (LSF) in the scanning direction, derived from the first-order derivative of the ESF. The full-width at half-maximum (FWHM) resolution was measured to be ~80 μm.

### 3.3. Performance of Photoacoustic Probe

**Figure 5:**
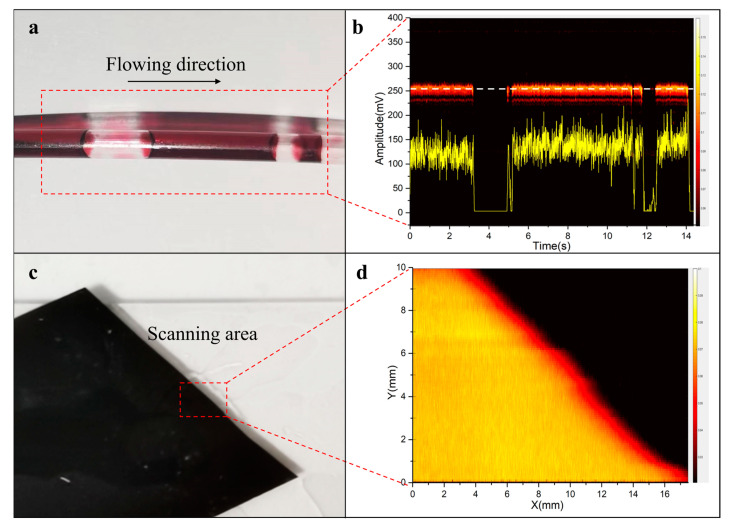
Photos and photoacoustic sensing/imaging results of phantoms. (**a**) Photo and (**b**) photoacoustic signals of red ink in tubing; (**c**) Photo and (**d**) photoacoustic MAP image of a black tape.

Figure 5 shows the sensing and imaging performance of the photoacoustic probe. Figure 5a,c show the photos of phantoms including the red ink in the PE tube and the black tape. Figure 5b presents the signals of flowing red ink, in which the yellow curve is the signal amplitude profile at the white dashed line of the B-scan photoacoustic signal with time. To match the flow speed (20 mm/s), the laser irradiation rate was set to 250 Hz based on the lateral resolution of ~80 μm. Figure 5b contains a 14 s dataset (3500 A lines) that took 0.68 s to process the raw data in Matlab and display the B-scan image. The result showed that the probe could distinguish the air bubbles from the flowing ink, being capable of discerning whether the fluid flow is continuous. Figure 5d shows the maximum amplitude projection (MAP) image of the black tape, showing the imaging capability of the probe.

## 4. Discussion and Conclusions

The miniaturization and simplification of devices are conducive to clinical implementation. The reported transparent photoacoustic probes were mostly with centimeter-scale dimensions, limiting their applications in the limited space. As the smaller LN wafer may only help to reduce the aperture size but likely degrade the transducer performance such as sensitivity, the issue can only be overcome via modifying the probe design and fabrication process. Here, we compare the performance of the proposed TUT with the previous studies using the same active material in Table 1, demonstrating its strengths on the dimension and center frequency. Its bandwidth and optical transmission efficiency are also comparable to the reported values. Details of the test method and the result of optical transmission efficiency are shown in the Supplementary Material.

The ITO thickness should be set in a reasonable range to balance the transparency and the electrical resistance. Different thicknesses of ITO were deposited and studied in terms of electrical properties. The thickness of 150 nm was optimized. To achieve a relatively low sheet resistance (~40 Ω ohm·cm), the sputtering process was operated at low power for a long duration.

To further shrink the probe size, we propose to combine the GRIN lens and the LN wafer as a whole. The GRIN lens is a key element for beam shaping. Epoxy was used as adhesion between the lens and LN wafer. The air bubbles in the epoxy could be removed by pressing the GRIN lens gently on the fragile wafer before curing.

There are two major types of optical fiber applied for laser delivery: the SM fiber offers a smaller spot while the multi-mode fiber offers higher laser energy. Both have the same packaging, so we chose the SM fiber with the pigtailed ferrule that was easy to align with the GRIN lens coaxially with the ferrule sleeve. With the proposed design, the fiber could be changed easily to meet various demands of applications.

The miniature probe was designed for multiple applications, especially in the limited space. For example, an ideal flow cytometer is portable for placing on the skin to detect the blood and cells in the subcutaneous vessel. The probe should be small and light enough to be worn on the hand or arm. To evaluate the performance of flow sensing, the red ink and tube were used to mimic the blood and the vessel. The signals of red ink and air bubbles could be easily identified (Figure 4b), while for the dynamic imaging, the probe could image the edge of the black tape clearly as PAM. All the results suggested that the miniature probe was capable of performing different functions such as fluid flow sensing or photoacoustic imaging.

Though the results show the feasibility and potential of the proposed photoacoustic probe, there is still room to further improve the performance. (1) As a miniature transducer, the bandwidth was too narrow, though this is a common issue due to the limited option of transparent materials for matching and backing layers. To retain the transparency of the TUT, parylene was not deposited. The multi-matching layer scheme should be studied and applied on the TUT to improve the bandwidth. (2) The GRIN lens could not absorb the ultrasound from the back of the LN wafer such that the echo signals and corresponding images would be adversely affected by ultrasound reflections. A longer GRIN lens may shift the unwanted reflections out of the ultrasound imaging range. (3) The mechanical scanning speed limits the potential of the probe, especially for the application of PAM. Optical scanning could speed up the imaging, which should be studied in the future.

To summarize, a miniature photoacoustic probe based on a 4 mm diameter TUT was successfully developed, whose dimension was smaller than that of the reported LN-based transparent ultrasound transducer. For the first time, the GRIN lens was used as the backing layer of the TUT, effectively shrinking the probe size. The probe was tested and found to have a high center frequency of 46.9 MHz and a −6 dB bandwidth of 29.4%. Experiments were further conducted on the mimicking phantom and black tape to show the potential in the fluid flow sensing and imaging applications, respectively.

## Figures and Tables

**Table 1:** Comparison of TUT parameters in different works.

Material	Wafer Size (mm)	Probe Diameter (mm)	Center Frequency (MHz)	−6 dB Bandwidth (%)	Optical Transmission Efficiency (%)	Reference
LN	2.5 × 2.5 and 10 × 10	N/A	14.5	30	~80 (690–970 nm)	[10]
LN	10×10	15	36.9	33.9	90 (Visible range)	[11]
LN	7×7	14	11.2	23	66 (690–910 nm)	[26]
LN	Φ9	9	7.5, 31.5	N/A	74 (630 nm)	[15]
**LN**	**2 × 2**	**4**	**46.9**	**29.4**	**57–89** (450–1200 nm)	**This work (Details in S2)**

## Data Availability

Data underlying the results presented in this paper are not publicly available at this time but may be obtained from the authors upon reasonable request.

## Supplementary Materials

The following supporting information can be downloaded at: https://www.mdpi.com/article/10.3390/mi14061269/s1, Figure S1: Optical transmission efficiency of LN wafer in the range of 450–200 nm; Figure S2: Result of long-term flow sensing; Figure S3: Data processing flowchart of photoacoustic imaging; Figure S4: Absorption spectrum of red ink.
