# Supplementary material for: A Miniature Multi-Functional Photoacoustic Probe
Source: Micromachines (Basel). 2023 Jun 19;14(6):1269. doi: 10.3390/mi14061269 (PMC10304571; doi:10.3390/mi14061269)
Supplement: Supplementary file 1 [file micromachines-14-01269-s001.zip › micromachines-2413066-supplementary.pdf]

Supplementary material

S1. Optical transmission efficiency of LN wafer.

To test optical transmission efficiency of LN wafer, a pulsed OPO laser (EKSPLA, NT242, Lithuania) was applied to offer the energy. A laser energy meter (PE9-C, Ophir, Israel) was used to measure the incident and transmitted light at the wavelength interval of 25 nm. The result (shown in Supplementary Figure S1) suggests that the LN wafer used in the TUT has over 60% of optical transmission from 450 nm to 1200 nm, which is suitable for various applications of multi-wavelengths.

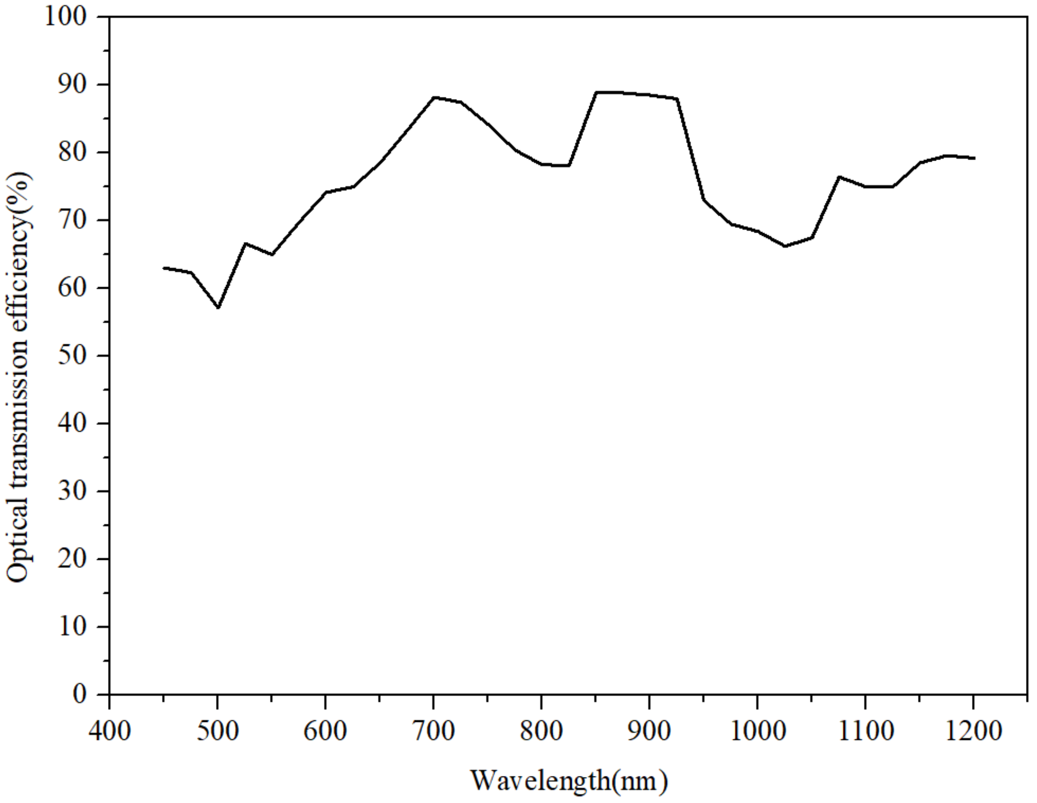

Supplementary Figure S1. Optical transmission efficiency of LN wafer in the range of 450–200 nm.

S2. Long-term stability of the probe

To evaluate the long-term stability of the proposed probe, the test was conducted using flow sensing. Red ink in a polyethylene tube was used to mimic the blood. As the blood circulation time in human body is ~20 seconds, the setup of red ink flow for 20 minutes mimicked ~60 cycles of blood circulation. The irradiation laser was with the wavelength of 532 nm, the repetition rate of 10 Hz, and the energy of 1 μJ. The photoacoustic signal of red ink was then recorded and processed. Supplementary Figure 2 exhibits the maximum amplitude of each A-line in every cycle, showing the fluctuation of the signal detection. The average of the data was 0.0941, while the standard deviation was 0.0054. According to the equation:

$$Coefficient\ of\ variation = \frac{Standard\ deviation}{Mean}$$

The coefficient of variation is ~5.7%, showing a good long-term detection stability of the probe.

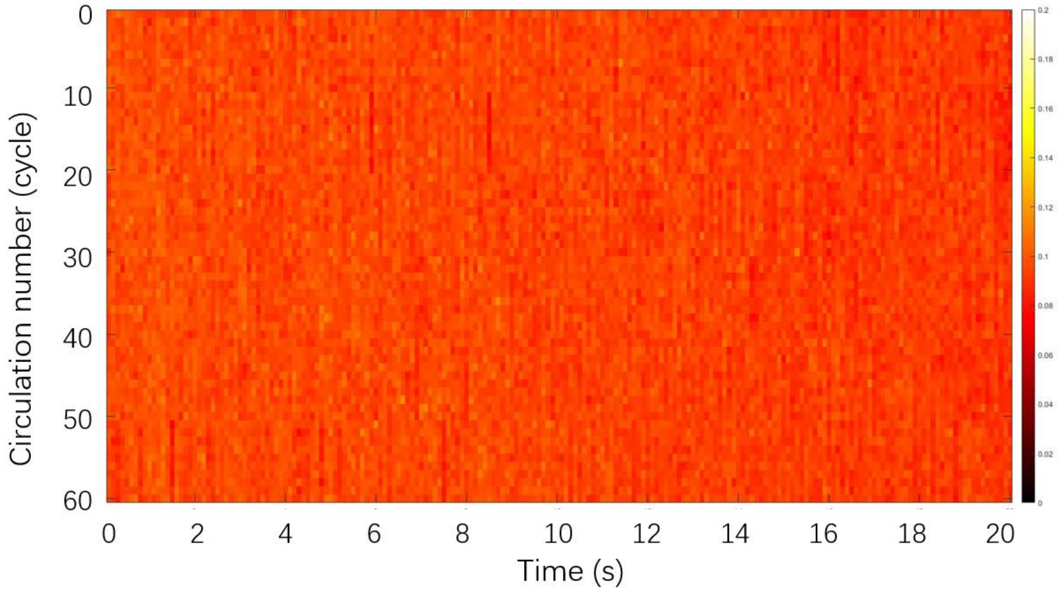

Supplementary Figure S2. Result of long-term flow sensing.

S3. Details of image processing

The photoacoustic signal was detected by the probe and amplified using an ultrasound pulser/receiver (5073PR, Olympus, Japan), digitized via a 1 GHz/s data acquisition (DAQ) card (ATS9371, Alazar, Canada), and then stored in a personal computer. The imaging data was processed following the flowchart (Supplementary Figure 3) as below. The raw data was input to program, and denoised by band-pass filtering. Then the amplitude envelope of each signal was extracted by the Hilbert transform. The B-scan image was obtained by arranging all the A-lines in time sequence. The maximum amplitude of each A-line was projected onto the two-dimensional x-y plane to get an MAP image.

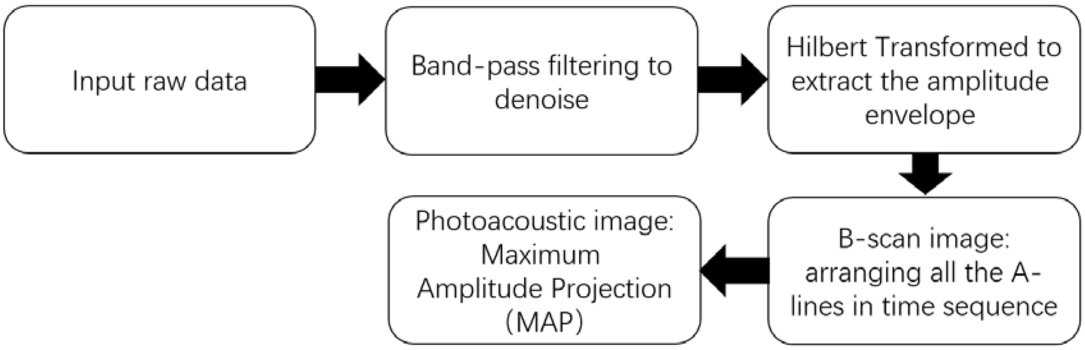

Supplementary Figure S3. Data processing flowchart of photoacoustic imaging.

S4. Absorption spectrum of phantom

To show the design rationale of the phantom, the absorption property of the red ink was tested in the spectrophotometer. Supplementary Figure 4 shows the absorption spectrum of red ink. The absorption peak is at 521 nm, which is near the wavelength of laser (532 nm) employed in the present work.

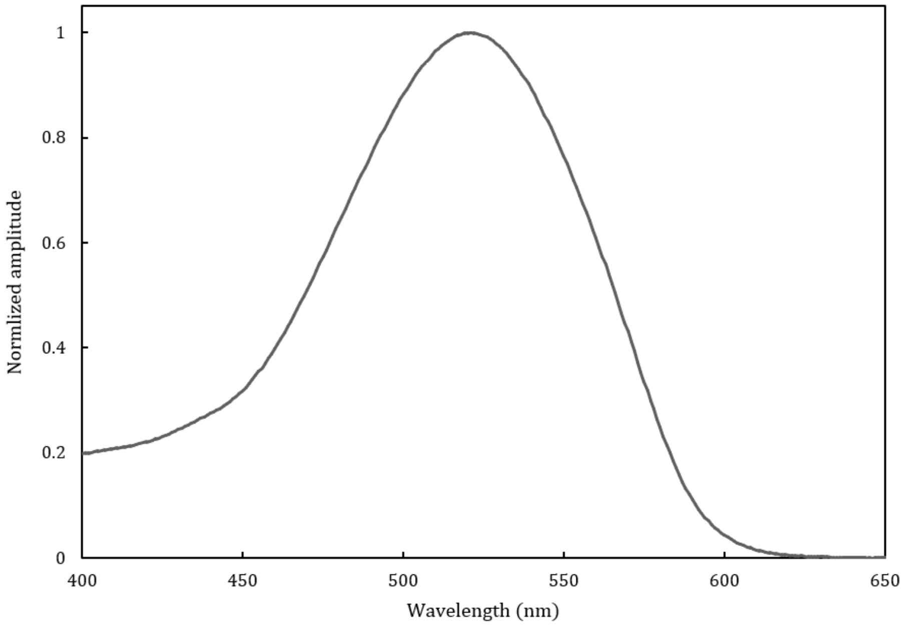

Supplementary Figure S4. Absorption spectrum of red ink.
